# Biphasic adverse effect of titanium nanoparticles on testicular function in mice

**DOI:** 10.1038/s41598-019-50741-9

**Published:** 2019-10-07

**Authors:** Nobuhiko Miura, Katsumi Ohtani, Tatsuya Hasegawa, Hiroki Yoshioka, Gi-Wook Hwang

**Affiliations:** 1 Industrial Toxicology and Health Effects Research Group, Japan National Institute of Occupational Safety and Health, 6-21-1 Nagao, Tama-ku, Kawasaki, Kanagawa pref. 214-8585 Japan; 2 Division of Human Environmental Science, Mount Fuji Research Institute, Yamanashi Prefectural Government, 5597-1 Kenmarubi, Kamiyoshida, Fujiyoshida, Yamanashi pref. 403-0005 Japan; 3 College of Pharmacy, Kinjo Gakuin University, 2-1723 Omori, Moriyamaku, Nagoya, Aichi 463-8521 Japan; 4 Laboratory of Molecular and Biochemical Toxicology, Graduate School of Pharmaceutical Sciences, Tohoku University, 6-3 Aoba Aramaki, Aoba-ku, Sendai, Miyagi-pref. 980-8578 Japan

**Keywords:** Metals, Mechanism of action

## Abstract

The male reproductive system is being recognized as toxic targets of nanoparticles including titanium dioxide nanoparticles (TiNP). Most of these reports are, however, obtained from the results of long-term exposure of TiNP. In this study, we diversely examined the acute effects of TiNP on the male reproductive system. Male C57BL/6J mice were administered a single intravenous injection of TiNP (10, 50 mg/kg), and were sacrificed at 1, 3, and 9 days post-injection. Testicular functions (estimated by sperm motility and sperm number) were measured via computer-assisted sperm analysis (CASA). Results indicated that sperm motility was significantly reduced from 1 day following TiNP injection (in both dose), and this reduction persisted up to 9 days post-TiNP injection (10 mg/kg injection group). Interestingly, we observed no significant decrease in sperm numbers in both the testis and the cauda epididymis in either treatment groups during the course of the experiment. Therefore, we hypothesized that TiNP may target the mature spermatozoa. In addition, sperm suspensions directly incubated with TiNP showed reduced sperm motility, [^3^H]-thymidine incorporation, and ATP level. Our results indicated that TiNP possesses “biphasic effects”; the obstacles to mature sperms (short term effect) in addition to the impairment in testis (long-term effect).

## Introduction

Titanium dioxide nanoparticles (TiNP) are used in a broad range of applications and consumer products such as exterior wall paints, antibacterial agents, white pigments, and sunscreens^1–3^. TiNP possess various physico-chemical characteristics, including self-cleaning, antibacterial, photocatalytic, and ultraviolet protection properties^4^. In Japan, the handling amount (sum of productions and imports) of TiNP is the third largest, after carbon black and silica (in “Nanomaterials Information Sheet” by Ministry of Economy, Trade and Industry, Japan). It is important that factories involved in the manufacturing of TiNP products handle raw TiNP materials. Therefore, we need to consider the health effects of TiNP on these factory workers. Furthermore, TiNP is widely used as a food additive in food industries. Considering the intake of various materials from food products, oral intake is one of the major route of TiNP. There is an interesting report that 75 kg adult human receive 15–37.5 mg/kg/day from food^5^. Therefore, we also need to pay attention to the health effects of TiNP on the general public, in addition to factory workers. As compared with that of traditionally used titanium fine particles, TiNP have a larger surface area to volume ratio; hence, TiNP may pose potential health risks to humans. With the rapid progression of nanotechnology, increasing attention has been placed on the potential hazardous effects of TiNP on health.

Although TiNP has been viewed as a nanoparticle with poor solubility and low toxicity^6^, many studies have observed TiNP-induced toxicity in the liver^7^, the lung^8^, the intestine^9^, as well as the central nervous system^10^. We have previously reported that the testes are susceptible to titanium toxicity, and that the male reproductive system is a TiNP health risk target^11,12^. Furthermore, several reports indicated that TiNP-induced testicular dysfunction can be induced via various injection routes such as intragastric^13^, intraperitoneal^14^, and intravenous injections^12^. It has been shown that TiNPs are taken up into Leydig cells, and inhibit the viability and proliferation of these cells^15^. Furthermore, TiNPs disrupt the mouse blood-testis barrier by altering the expression of several testis-specific genes^16^. These results confirmed that the testis is a target of TiNP.

Most of these reports, however, only examined the effect of long-term TiNP exposure. Recent studies have also begun to explore the short-term effects of TiNPs exposure. For example, one study investigated TiNP (anatase form)-induced sperm defects at 24 to 120 h post-injection (administered intraperitoneally for three consecutive days)^14^. However, some studies found no obvious male reproductive disorders in mice at 1 and 7 days post-injection (administered a single dose of TiNP intravenously)^17^. Therefore, the short-term impact of TiNPs is not clear. We have also noticed the possibility that acute testicular dysfunction occurred clearly even for short-term administration of TiNP. In this paper, we diversely examined the acute effects of TiNP on the male reproductive system.

## Results

### Acute effect of TiNP on testicular function

**Figure 1:**
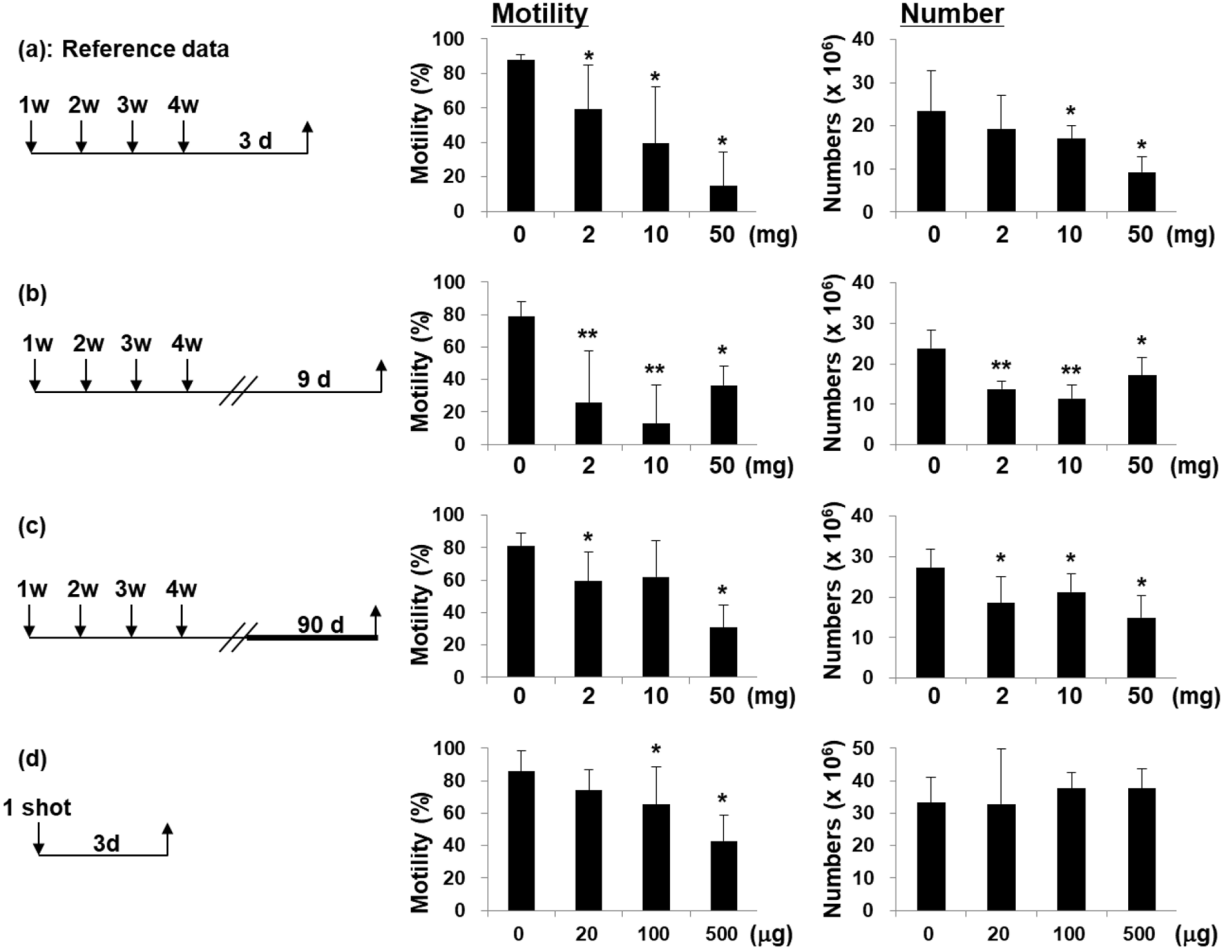
Acute effect of TiNP on testicular function. Male C57BL/6J mice (n = 5) were injected with TiNP (2, 10, 50 mg/kg; i.v.) once a week for 4 weeks, and were sacrificed at 3 days (**a**); 9 days (**b**); or 90 days (**c**) after the final injection. Control mice received DSP (0.1 ml/20 g body weight). Mice received single IT injection of TiNPs (20, 100, or 500 μg/mouse), and were sacrificed 3 days later (**d**). Sperm motility (motility percent) and sperm numbers (in cauda epididymis) are shown. Results from (**a**) was derived from a previous paper. In two experiments (**b**,**c**), C57BL *gpt* delta mice were used. *, **, significantly different from control (*p < 0.05; **p < 0.01).

We have previously reported that TiNP decreases both sperm motilities and sperm numbers in mice i.v.-injected with TiNP once a week for 4 weeks; testicular function was examined 3 days after the final injection (Fig. 1a, as a reference data)^12^. In subsequent studies, testicular dysfunction (reductions in both sperm motilities and sperm numbers) was observed not only after 9 days (Fig. 1b), but also after 90 days from date of last TiNP administration (once a week for 4 weeks, i.v.) (Fig. 1c). However, we noticed that sperm motility declined significantly 3 days after only one injection of TiNP, although the sperm number did not decrease (IT injection; Fig. 1d). In mice, it is known that the duration of spermatogenesis (from spermatogonia to mature spermatozoa) is approximately 35 days^18^. Therefore, the decrease in the sperm number observed in the experiment groups at 4 weeks following the first injection (Fig. 1a–c) was thought to be due to attack on spermatogonia cells in the testis. In the one-shot experimental group (Fig. 1d), however, only sperm motility was decreased. Therefore, we hypothesized that TiNP may attack mature spermatozoa.

**Figure 2:**
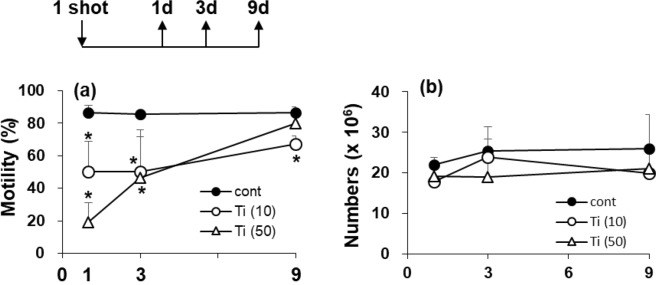
Temporal change in testicular indexes following TiNP injection. Male C57BL/6J mice received a single i.v. injection of TiNP (10, 50 mg/kg), and were sacrificed at 1, 3, and 9 days post-injection. Control mice received DSP (0.1 ml/20 g body weight). Sperm motility percent (**a**) and sperm numbers in the cauda epididymis (**b**) were indicated. *, **, significantly different from control (*p < 0.05; **p < 0.01).

In order to investigate this possibility, we conducted a short time course experiment following a single i.v. injection of TiNP into mice. A significant reduction in sperm motility was observed in the 10 mg/kg injection group the next day; the decreased sperm motility was maintained until 9 days after the injection (Fig. 2a). In the 50 mg/kg administration group, a marked decrease in sperm motility was also observed 1 day after the injection. However, sperm motility was increased 3 days after the injection; at 9 days post-injection, the value was comparable with that of the control group (Fig. 2a). Interestingly, we did not observe any significant change in the sperm numbers of the cauda epididymis during the course of the experiment (Fig. 2b). We observed no noteworthy influence of TiNP injection on body weights and organ weights of the testes, the epididymis, and the cauda epididymis (data not shown). Further, since histological data of the testis from the “Reference data” (Fig. 1a) showed no noticeable impairments by TiNP injection, we considered that testicular dysfunction by this administration method of TiNP was not caused by an organic disorder. Therefore, we did not perform histological analysis even in this short-term exposure.

### Direct effect of TiNP on mature spermatozoa

**Figure 3:**
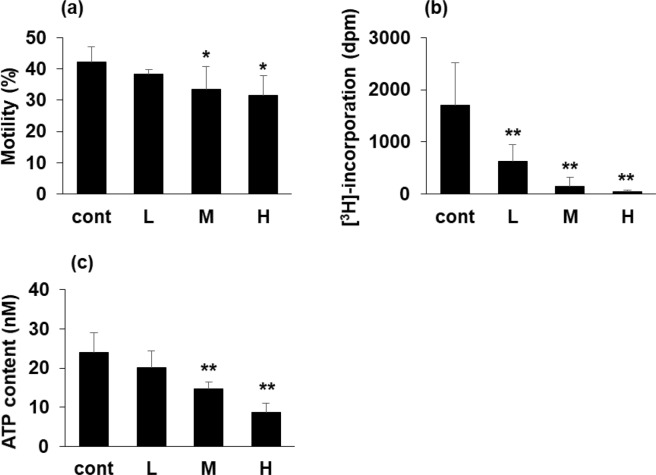
Direct effect of TiNP on mature spermatozoa. Sperm suspensions (1 × 10^4^/well, n = 4) obtained from the right cauda epididymis were dispensed into 96 well plates. Sperm suspensions were treated with TiNP (2.19, 4.38, and 8.76 ng/μl; shown as L, M, H, respectively). Sperm motility was measured 3 h post-treatment (**a**). For [^3^H]-thymidine incorporation analysis, sperm suspensions (1 × 10^4^/well, n = 4) treated with TiNP were also incubated with 1.85 kBq of [^3^H]-thymidine for 3 h. Sperm lysate was dripped onto the glass filter (GA-100) to adsorb sperm DNA. Radioactivity of the glass filter was quantified by a liquid scintillation counter (**b**). To measure sperm ATP content, sperms treated with TiNP as in (**a**) were collected by centrifugation. Sperm ATP levels were determined using a luciferase-based IntraCellular ATP assay kit (**c**). *, **, significantly different from control (*p < 0.05; **p < 0.01).

We examined the effect of TiNP on isolated mature spermatozoa. Sperm suspension obtained from the cauda epididymis was directly incubated with TiNP for 3 h. Results indicated that sperm motility was decreased by TiNP treatment in a dose-dependent manner (Fig. 3a). Since the activity of cultured cells can be examined by incorporation of [^3^H]-thymidine into DNA, we thought that sperm activity could also be checked by [^3^H]-thymidine incorporation as well. With reference to the report that spermatozoa from ram can incorporate [^3^H]-thymidine into their mitochondrial DNA^19^, we examined the [^3^H]-thymidine incorporation level after TiNP addition. The level of [^3^H]-thymidine incorporation was also markedly decreased by TiNP treatment in a dose-dependent manner (Fig. 3b). These results suggested that TiNP may exert direct effects on sperm function.

It is well understood that ATP synthesized in the mitochondria is important for sperm motility^20^. Therefore, we also examined the effect of TiNP on sperm ATP content. As a result, TiNP induced a dose dependent reduction on ATP content in isolated sperms (Fig. 3c). As one information, we have a data from *in vivo* experiment that showed similar result as an *ex vivo* experiment described above. Because this *in vivo* experiment was only performed on one animal (n = 1) due to technical reason (while dissecting the mice, it was difficult to measure the ATP in the desired time period from the sperm suspension), we described here only as a reference (data not shown). Furthermore, since the decrease in ATP level suggested a mitochondrial functional defect, we considered examining the mitochondrial membrane potential in sperm in the future.

### Testicular dysfunction by oral administration

**Figure 4:**
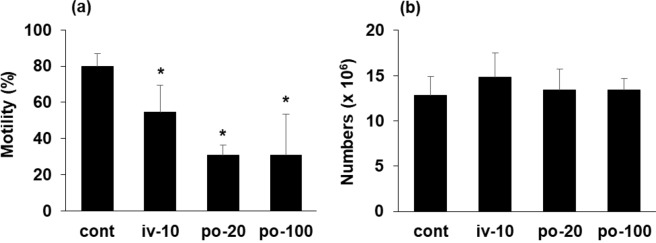
Testicular dysfunction by oral administration. Male C57BL/6J mice were orally administered with a single shot of TiNP (20 or 100 mg/kg, shown as “po”). As a positive control, one group mice were received a single shot of i.v. injection of TiNP (10 mg/kg). Testicular function was examined 3 days post-TiNP administration. Control mice were administered DSP (0.1 ml/20 g body weight). Sperm motility percent (**a**) and sperm numbers in the cauda epididymis (**b**) were determined. *, **, significantly different from control (*p < 0.05; **p < 0.01).

In this paper, we chose intravenous injection as a route of injection, since this method ensures the TiNP entry into the blood. Considering the use of TiNP and the route of exposure, however, it is usually taken in orally in the case of food additives. TiNP exposure in workers that handle nano-sized titanium as raw materials in factories is usually achieved via inhalation and oral exposure. Furthermore, one report estimated that the daily intake of TiNP for adults is 15–37.5 mg/kg from food^5^. Therefore, we compared the extent of testicular damage caused by oral administration (20 or 100 mg/kg) of TiNP. Mice were orally administered with a single shot of TiNP; testicular function was examined 3 days following TiNP administration. Significant decrease in sperm motility was observed following oral TiNP administration, similar to that observed during i.v. (10 mg/kg) TiNP administration (Fig. 4a). However, the number of spermatozoa in the cauda epididymis remained unchanged (Fig. 4b).

## Discussion

Our present study showed that TiNP may directly attack mature spermatozoa, since short-term injection of TiNP efficiently inhibited mature sperm motility without affecting the sperm number in both the testis and the cauda epididymis. Increasing number of studies have shown that long-term repeated administration of TiNP induces testicular dysfunction (reduction in sperm numbers and sperm motility)^12–16,21^. Therefore, the results of our present study revealed that TiNP possesses “biphasic effects” that include impairments to the testis (long-term influence)^14,21^ as well as the mature sperm (short-term influence).

It is known that the duration of spermatogenesis, from spermatogonia to mature spermatozoa, is approximately 35 days in mice^18^. Therefore, the reduction in sperm numbers and sperm motility in the experimental groups 4 weeks following the first injection (Fig. 1a–c) was likely due to attack on spermatogonia cells in the testis. However, the reduced sperm motility in one the shot group (Figs 1d and 2) was due to direct attacks on mature spermatozoa in the cauda epididymidis.

**Figure 5:**
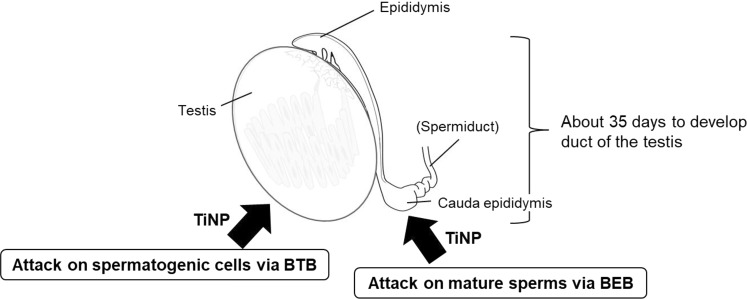
Schematic of the biphasic adverse effect of TiNP on testicular function.

Reactive oxygen species (ROS) is thought to be a triggering mechanism of testicular dysfunction following exposure to nanosized materials including titanium^14,22^, as ROS can affect many kinds of molecules (e.g., nucleic acids, proteins, and lipids). Smith *et al*. reported that short-term exposure to TiNP (anatase form) significantly increases ROS level, resulting in structural sperm defects^14^. In addition, as evidenced by changes in cytokine status, anatase titanium can bypass the blood-epididymis barrier to enter the epididymal lumen, which indicated that TiNP has the ability to traverse biological barriers^14^. TiNP are taken up into Leydig cells and inhibit the growth and proliferation of these cells^15^. Furthermore, TiNP disrupts the mouse blood-testis barrier by altering the expression of several testis-specific genes^16^. In our one-shot experiments, we only observed a decrease in sperm motility with no change in sperm number (Figs 1d, 2 and 5). Therefore, we believe that the influence on TiNP on mature sperm in cauda epididymis is immediate, although the effect of TiNP on spermatogonia cells in testis does not appear as a phenotype (sperm number reduction) until 30 days or later. Our data indicated that acute TiNP exposure induces detrimental effects on mature spermatozoa in the cauda epididymis.

In our time course experiment with a single i.v. injection of TiNP, the lower dose (10 mg/kg) group exhibited a significant decrease in sperm motility from day 1 to day 9 after TiNP administration (Fig. 2a). The higher dose (50 mg/kg) group only showed a transient decline in sperm motility, with the lowest point at 1 day post-injection (Fig. 2b). At higher TiNP dose, the capillary vessels (and/or blood-epididymis barrier^14,23^) may become blocked owing to extensive aggregation, and the blood supply containing nanoparticles was not sustained. As a result, testicular dysfunction was allowed to recover. Conversely, in the lower dose (10 mg/kg) group, vessels and/or barrier were less likely to become blocked, and the effect of TiNP (induction of testicular dysfunction) was sustained. Our results showed that even a small amount of TiNP may lead to sustained biological effects (in our case, the testicular dysfunction). In the testis and the cauda epididymis, Ti levels measured using ICP-MS with the mass number of 47 m/z were below detection limit (not detected). It is considered that quantitative analysis is impossible under this administration condition. We prepared the sections of the cauda epididymis and are currently conducting SEM analysis of the state of Ti in the section.

Since the level of [^3^H]-thymidine incorporation in spermatozoa was markedly reduced following TiNP treatment (Fig. 3b), we hypothesized that reduction in sperm motility by TiNP was due to its direct effect on mature sperms. Further, TiNP reduced the ATP content in collected sperms (Fig. 3c). Preliminary data indicated that TiNP inhibits ATP-citrate synthase, a transferring enzyme involved in the synthesis of ATP by converting acetyl-CoA to CoA^24^. It is possible that ATP-citrate synthase is one of the targets of TiNP. Moreover, TiNP may inhibit ATP synthase, resulting in reduced ATP content, which leads to compromised motor functions in the sperm. Further studies using comprehensive methods, such as microarrays, are still needed to determine the mechanisms underlying TiNP-induced testicular dysfunctions, including the inhibitory effect of TiNP on ATP-citrate synthase.

TiNP-induced testicular dysfunction (inhibition of sperm motility and reduction of sperm number) is a clear phenomenon. The influence of TiNP exposure on fertilization needs to be investigated in the future. Middle- to long-term exposure of TiNP induces significant defects, both a qualitative change that decreases sperm motility as well as a quantitative change by reducing the sperm number. The TiO_2_ (Aeroxide-P25) used in this paper exhibited mixed crystallinity in the anatase form as the predominant form (80% anatase: 20% rutile). The rutile type is considered to have low bioactivity, and is used for several nano-products such as food additives and pharmaceutical excipients. On the other hand, the biological activity of the anatase form with photocatalytic activity is thought to be high, and causes testicular disorders. Therefore, it is necessary to clarify which TiO_2_ type induces testicular dysfunction. We compared rutile form and anatase form with respect to sperm motility inhibitoion, and obtained the perliminary result that anatase type shows the testicular dysfunction. The effect of various TiO_2_ crystal form on testicular dysfunction (using sperm number, sperm motility, and fertility as indicators) is an important point of consideration in future use of nano-sized titanium.

In terms of occupational health, our results are important in consideration of acute TiNP exposure due to unexpected accidents (natural disasters such as earthquakes, leakage accidents, explosion accidents) in industries that handle TiNP as raw materials. The safety data sheet (SDS) of titanium dioxide does not contain any data on reproductive toxicity. As mentioned above, TiNP induces testicular dysfunction (decreased both sperm number and motility) upon medium- to long-term exposure. Moreover, it induces acute injury (decreased sperm motility) upon short-term exposure. Results from the current study can be used as basic data to evaluate the reproductive toxicity of TiNP. We found that TiNP induces similar testicular dysfunction via multiple exposure routes (see Figs 1 and 4). In particular, testicular dysfunction by oral administration is an important issue to consider, since TiNP will enter the body from the oral route when breathing. Therefore, it is necessary to consider the health effects of TiNP through both inhalation and oral exposure in industries that handle nanomaterials. Moreover, under general environment, oral exposure is considered to be a more general route than inhalation exposure, since TiNP is used as a nano-product (such as food additives, pharmaceutical excipient, etc.) rather than nanomaterials. A Monte Carlo human exposure analysis to TiNP reported that a typical exposure for a US adult may be on the order of 1 mg/kg/day^25^. Importantly, this report identified that children (under the age of 10 years) received the highest exposure (1.5 to 2 mg/kg/day) than that of consumer age groups, because: 1) TiNP content of sweets (as for white color pigment) is higher than other food products; and 2) the consumption of sweet products is relatively is relatively large compared to adults. Although there is an interesting report that 75 kg adult human receive 15–37.5 mg/kg/day from food^5^, it is considered that further studies will be necessary at lower doses in order to understand the biological effects of TiNP more accurately, including the effects on children. Our findings contribute to a basic understanding of TiNP that is valuable for further investigations into the reproductive toxicity of TiNP.

In this paper, we found that TiNP possesses “biphasic effects”, which includes impairment of the testis via blood-testis barrier (BTB) attacks (long-term influence)^14,21^, as well as damages to mature sperms via blood-epididymis barrier (BEB) attacks (short-term influence) (Fig. 5). In terms of occupational health, our results are important when considering acute exposure to TiNP due to unexpected accidents (natural disasters such as earthquakes, leakage accidents, explosion accidents,) in industries that handle TiNP as raw materials. There are currently no data available on the reproductive toxicity of titanium dioxide in the SDS. As mentioned above, TiNP induces testicular dysfunction (decreased both sperm number and motility) upon medium- to long-term exposure, and also induces acute injury (decreased sperm motility) upon short-term exposure. Therefore, considerations need to be made regarding the reproductive toxicity of TiNP.

## Methods

### Preparation of TiNP suspension

TiNP suspension was prepared as previously described^12^. Briefly, titanium dioxide (Aeroxide-P25) purchased from Sigma-Aldrich (St. Louis, MO, USA) was sterilized by a dry heat sterilizer (180 °C for 1 h). The powder was suspended in sterile disodium phosphate (DSP; 2 mg/ml) in a glass vial at a concentration of 10 mg/ml. The TiNP suspension was sonicated in an ultrasonic water bath (Bransonic 2510; Branson, Danbury, CT, USA) for 30 min. For the vehicle control, sonicated DSP was also prepared. The zeta potential of TiNP after sonication was determined using the particle and molecular size analyzer, Zetasizer Nano-ZS (Malven, Worcesterchire, UK). The Z-average of the TiNP was approximately 150 d.m.^26^.

For *ex vivo* analysis, sterilized TiNP (Aeroxide-P25) was suspended in ultrapure water (100 mg/ml), sonicated for 30 min, and centrifuged at 1,000 × *g* for 60 min at room temperature (r.t.). The supernatant was filtered using a 0.45 μm filter. Ti concentration in the resultant suspension was measured using inductively coupled plasma mass spectrometry (ICP-MS, Agilent 7900, Agilent Technologies Japan) at a mass number of 47 m/z. The stock concentration was 87.6 ng/μl.

### Experimental procedures

Male C57BL/6J mice (8 weeks) were purchased from Clea Japan (Tokyo, Japan). Male C57BL/6J *gpt* delta transgenic mice, used in our previous work^26^ to assess the genotoxicity of TiNP, were purchased from JapanSLC (Shizuoka, Japan). Mice were kept in cages under standard conditions with controlled temperature (24 ± 1 °C), humidity (55 ± 5%), and light (12:12 h light/dark cycles, lights on at 08:00 AM). All animals were fed sterilized commercial pellet diet (CE-2, Clea Japan, Tokyo, Japan) and filtered tap water *ad lib*. Mice were allowed to acclimatize for at least 7 days prior to experimentation. For both intravenous (i.v.) and per oral (p.o.) administrations, mice were administered TiNP suspension (0.1 ml/20 g body weight) diluted to the desired concentration using DSP. Control mice were also administered with DSP (0.1 ml/20 g body weight). For intratracheal (IT) administration, TiNP was suspended in sterilized distilled water and filtered (0.22 μm). Mice received IT injections (40 μl/mouse) at 20, 100, or 500 μg TiNP/mouse using a 27-gauge needle attached to a microliter syringe^27^. Following an appropriate period from each administration, mice were sacrificed under carbon dioxide anesthesia. The testicular organs (testes, epididymis, and cauda epididymis) were isolated and weighed. The right cauda epididymis was immediately analyzed for sperm motility, and other organs were stored in the −80 °C freezer until analyses.

All animal experiments were carried out in strict accordance to the recommendations in the guidelines for the care and use of laboratory animals set forth by the Institutional Animal Care and Use Committee at the Japan National Institute of Occupational Safety and Health (JNIOSH) and were approved by the Committee for the Care and Use of Laboratory Animals at JNIOSH (permission number H27-03-4).

### Sperm motilities

Sperm motilities were measured as previously described^12^. In order to ensure reproducibility, we have decided to use the right side for measuring the sperm motility. Briefly, for preparation of sperm suspension, the right cauda epididymis was immerged into a 35 mm dish containing 2 ml M199 medium (Thermo Fisher Scientific, Waltham, MA, USA) and 0.5% bovine serum albumin (BSA). Tissues were minced with scissors to release spermatozoa into the medium. Following a 5 min incubation period at 37 °C in the CO_2_ incubator (5% CO_2_ in air), the sperm suspension was loaded into a capillary micro slide. Sperm motility was analyzed by computer-assisted sperm analysis (CASA) using the HTM-IVOS (Hamilton Thorne Inc. Beverly, MA, USA).

### Sperm numbers

Sperm numbers were counted as sperm head numbers, as previously described^12^. Briefly, after homogenizing the left cauda epididymis in saline, sperm heads were stained with a Hoechst-based dye (IDENT STAIN; Hamilton Thorne Inc.). Fluorescence of sperm heads under the ultra-violet beam were counted automatically using CASA with the RAT-IDENT mode. Data were expressed as total number of sperm heads per tissue sample.

### *Ex vivo* analysis

Sperm suspensions were obtained from the right cauda epididymis as described above (in the “Sperm motility” section), and their motilities were counted. Sperms with a motility rate of 80% or more were used for the experiment. To determine the sperm concentration, sperm suspension (3 μl) was added to 3% NaCl (27 μl) to inhibit sperm movement; sperm numbers were then counted using a hemocytometer. Spermatozoa (1 × 10^4^) were suspended in 90 μl of M199 medium containing 0.5% BSA in a 96 well plate. The Ti stock solution (87.6 ng/μl) was diluted by two-fold and was added to each well (10 μl). The final concentrations of Ti suspensions were 8.76, 4.38, and 2.19 ng/μl. Following addition of TiNP solution, sperm suspensions were incubated for 3 h at 37 °C in the CO_2_ incubator. After that, sperm motilities were measured by CASA.

### [^3^H]-thymidine incorporation of spermatozoa

Sperm suspensions (1 × 10^4^/well) dispensed into a 96 well plate were treated with TiNP (as described above) and incubated with 1.85 kBq of [^3^H]-thymidine for 3 h. To measure the radioactivity in sperms, sperm lysate was prepared by addition of 5% SDS for 15 min on ice. The lysate was dripped onto the glass filter (GA-100, Advantec, Tokyo, Japan) to adsorb sperm DNA, vacuumed, and washed with water 3 times. Radioactivity of the glass filter was quantified by the liquid scintillation counter (Tri-Carb A5110TR, PerkinElmer Japan, Yokohama, Japan).

### ATP assay

Sperm ATP levels were determined using a luciferase-based IntraCellular ATP assay kit (IC100; Toyo ink, Tokyo, Japan) according to manufacturer’s instruction. Briefly, after incubating the sperm suspension with TiNP (3 hours, as described above), sperm suspensions (1 × 10^4^/100 μl) were centrifuged at 1,000 × *g* for 5 min at r.t. The medium was then removed, and ATP Extraction Reagent (60 μl) was added to lyse spermatozoa and to extract ATP. The extracts (50 μl) were transferred to a 96 well white plate (Thermo Fisher Scientific), and Luciferin-Luciferase Solution (50 μl) was added. Light emission was immediately measured using a microplate reader (Infinite 200 PRO; TECAN Japan, Kawasaki, Japan).

### Statistical analysis

All data were expressed as mean ± standard deviation (SD), and were analyzed by one-way ANOVA. Statistical significance of differences between control and TiNP-treated groups was determined with Dunnett’s test. In all cases, p < 0.05 was considered to be statistically significant.
